# The Ventral Tegmental Area and Nucleus Accumbens as Circadian Oscillators: Implications for Drug Abuse and Substance Use Disorders

**DOI:** 10.3389/fphys.2022.886704

**Published:** 2022-04-27

**Authors:** Darius D. Becker-Krail, William H. Walker, Randy J. Nelson

**Affiliations:** Department of Neuroscience, Rockefeller Neuroscience Institute, West Virginia University, Morgantown, WV, United States

**Keywords:** ventral tegmental area, nucleus accumbens, SCN, circadian rhythms, reward, oscillator, molecular clock, extra-SCN

## Abstract

Circadian rhythms convergently evolved to allow for optimal synchronization of individuals’ physiological and behavioral processes with the Earth’s 24-h periodic cycling of environmental light and temperature. Whereas the suprachiasmatic nucleus (SCN) is considered the primary pacemaker of the mammalian circadian system, many extra-SCN oscillatory brain regions have been identified to not only exhibit sustainable rhythms in circadian molecular clock function, but also rhythms in overall region activity/function and mediated behaviors. In this review, we present the most recent evidence for the ventral tegmental area (VTA) and nucleus accumbens (NAc) to serve as extra-SCN oscillators and highlight studies that illustrate the functional significance of the VTA’s and NAc’s inherent circadian properties as they relate to reward-processing, drug abuse, and vulnerability to develop substance use disorders (SUDs).

## Introduction

Life on Earth has evolved to adapt to the 24-h periodic cycling of temperature and sunlight as the result of the planent rotating about its axis around the Sun. Over evolutionary time, the predictable daily cycles of light and dark have been internalized in the form of circadian rhythms (Pittendrigh, 1993). These endogenous, self-sustaining rhythms allow for optimal synchronization of physiological and behavioral processes with the external environment. Circadian rhythms are highly conserved across the kingdoms of life and are present in archaea, bacteria, plants, and animals (Maniscalco et al., 2014; Walton et al., 2022). Predictably, circadian rhythms confer increased fitness by reducing energy expenditure and allowing organisms to anticipate, adapt, and organize their biological processes and behaviors to appropriate times of the day. Well-known examples of mammalian circadian rhythms are body temperature, sleep-wake cycle, and patterns of hormone secretion (e.g., cortisol and melatonin), but also include complex processes such as cognitive function, attention, stress, mood, and reward.

In mammals, the circadian system is organized in a hierarchal fashion. At the top lies the suprachiasmatic nucleus (SCN), a bilateral set of nuclei in the anterior hypothalamus (Kalsbeek et al., 2006). In the absence of environmental cues, a highly coupled network of neurons and glia within the SCN generate and maintain rhythms of ∼24-h. However, SCN rhythmicity can be entrained in the presence of zeitgebers (i.e., time givers) ; Kumar Sharma and Chandrashekaran, 2005). Although other zeitgebers exist (e.g., food, sex, socialization, and even drugs of abuse), in mammals, light is the most potent zeitgeber. Light signals to the SCN by first activating a specialized type of cell within the retina termed intrinsically photosensitive retinal ganglion cells (ipRGCs). ipRGCs are a small portion of the larger class of retinal ganglion cells within the eye. However, due to their expression of the photopigment melanopsin, these cells are precisely photosensitive (Brainard et al., 2001; Berson et al., 2002). The melanopsin photopigment within ipRGCs is maximally sensitive to blue light (∼480 nm) and is minimally activated in the presence of longer wavelength red light (>600 nm) (Spitschan, 2019). Of note, light acting directly on melanopsin containing ipRGCs is not the only mechanism by which ipRGCs can become active. Indeed, ipRGCs also serve to integrate light information from rods and cones (Prayag et al., 2019). Once activated, ipRGCs propagate their neural signal *via* the retinohypothalamic tract (RHT) directly into the SCN. Notably, in mammals, light input into the eye is exclusive *via* the RHT. Enucleation prevents photoentrainment, demonstrating that, unlike some vertebrates, there are no functional extra-retinal photoreceptors (Nelson and Zucker, 1981). This monosynaptic RHT pathway results in the release of glutamate and pituitary adenylate-cyclase-activating polypeptide (PACAP) onto the SCN (Hannibal et al., 2000). Activation of the SCN *via* release of glutamate and PACAP results in a rise in intracellular Ca^2+^ and cyclic AMP (cAMP) levels and activation of downstream signaling cascades [reviewed in detail (Ashton et al., 2022)]. Ultimately, the transcription factor cAMP response element-binding protein (CREB) is activated that in turn binds and modulates transcription of the core clock genes *Per1* and *Per2* (Ashton et al., 2022). Specifically, light-induced upregulation of *Period* genes ultimately adjusts the core TTFL, shifting and aligning the phase of the clock with the external light/dark cycle (Albrecht et al., 1997; Shearman et al., 1997). The SCN then relays this light-induced temporal information throughout the brain (e.g., extra-SCN central oscillators) and periphery (e.g., peripheral oscillators) *via* autonomic, metabolic, and hormonal signals; thus, synchronizing and organizing rhythmic activity of the organism.

**FIGURE 1:**
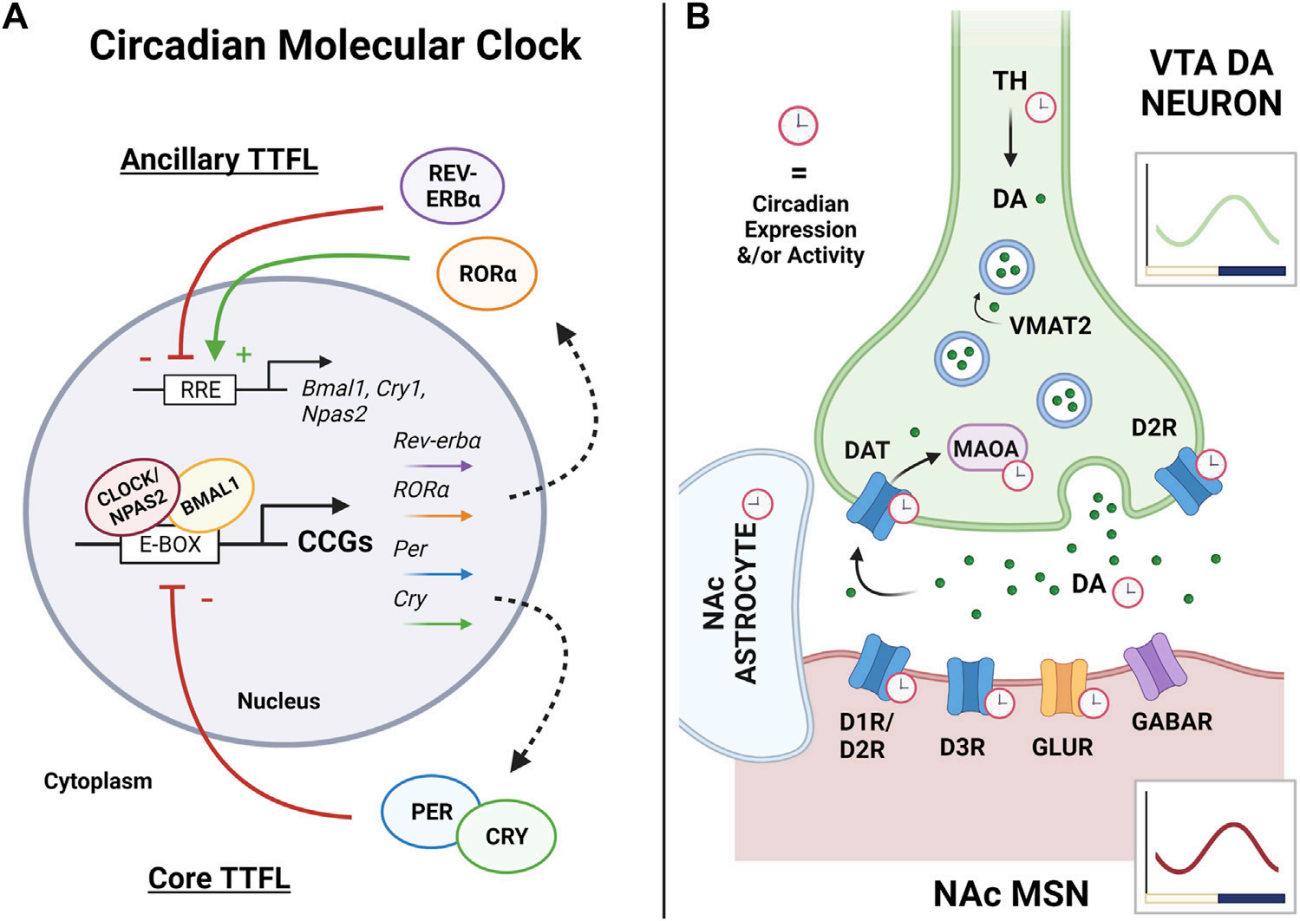
The circadian molecular clock and its regulation of the VTA-NAc synapse. **(A)** Rhythms of the circadian molecular clock are generated through a complex series of transcriptional-translational feedback loops (TTFLs). In the mammalian core TTFL, CLOCK (or its functional paralogue NPAS2) forms a heterodimer with BMAL1, binds to E-box elements in the promoter regions of DNA, and drives the transcription of many clock-controlled genes (CCGs), including *Period* and *Cryptochrome.* Throughout the day, PERs and CRYs accumulate in the cytoplasm, dimerize, and undergo phosphorylation. Into the night, PER:CRY dimers shuttle back into the nucleus and inhibit their own transcription through repressing CLOCK/NPAS2:BMAL1 activity, thus completing the core TTFL which cycles approximately every 24 h. Among its CCGs, CLOCK/NPAS2:BMAL1 also regulates the expression of the nuclear receptors *RORα* and *Rev-erbα* which form an ancillary TTFL of the molecular clock. RORα and REV-ERBα compete at Rev-erb/Ror response elements (RREs) in the promoter regions of *Bmal1*, *Npas2*, and *Cry1* to regulate their transcription, where RORα promotes expression and REV-ERBα represses. Along with others, this ancillary TTFL works to sustain, stabilize, and reinforce the core TTFL and rhythmic output as a whole, altogether temporally controlling nearly all aspects of cellular physiology. Arrows and (+) indicate promotion of expression, while bars and (−) indicate repression of expression. **(B)** The ventral tegmental area (VTA) and nucleus accumbens (NAc) circuit is the key reward pathway of the brain. Rewarding stimuli primarily stimulate dopamine (DA) neurons in the VTA to release DA into the NAc, driving medium spiny neuron (MSN) activity and subsequent reward-seeking behaviors. The circadian molecular clock has been shown to regulate nearly all components of DA synaptic transmission, with synthesis, uptake, and degradation all showing circadian variation in expression or activity. This includes the synthesis enzyme tyrosine hydroxylase (TH), the DA receptors (D_1_R, D_2_R, and D_3_R), DA release itself, the dopamine reuptake transporter (DAT), and the dopamine degradation enzyme monoamine oxidase A (MAOA). Overall rhythms in activity, molecular clock genes, and transcriptome wide rhythms have been detected in the VTA and NAc, with peaks aligning in the active phase (dark phase in nocturnal rodents). Robust rhythms have been detected in NAc astrocytes, as well as rhythms in GABA, glutamate, and glutamate receptors (GLUR) in the NAc. Red clocks indicate observed rhythms in expression or activity. Green dots are DA molecules. Vesicular monoamine transporter 2 (VMAT2) loads dopamine into the vesicles prior to synaptic release. Graphs illustrate representative wave form of overall VTA (green) or NAc (red) rhythmicity in nocturnal rodents, with yellow bar indicating light phase and purple bar indicating dark phase. Figure created with BioRender.com.

At the molecular level, the mammalian circadian system is driven by transcriptional-translational feedback loops (TTFLs; i.e., the “circadian molecular clock”), which are autoregulatory feedback loops of transcriptional activators and repressors (Figure 1A **)** (Mohawk et al., 2012; Takahashi, 2017). The proteins, brain and muscle arnt-like protein 1 (BMAL-1; encoded by *Arntl*) and circadian locomotor output cycles kaput (CLOCK), or the CLOCK paralogue neuronal PAS domain protein 2 (NPAS2), encompass the positive arm of the core circadian molecular clock (Vitaterna et al., 1994; Hogenesch et al., 1997; Ikeda and Nomura, 1997; King et al., 1997; Zhou et al., 1997; Hogenesch et al., 1998; DeBruyne et al., 2007). BMAL1 and CLOCK and/or BMAL1 and NPAS2 form heterodimers to bind to E-box regulatory elements within promoter regions of DNA to drive transcription of thousands of genes. Particularly relevant to the circadian molecular clock, these heterodimers drive transcription of the negative arm of the clock, namely, *Period* (*Per1*, *Per2* and *Per3*) and *Cryptochrome* (*Cry1* and *Cry2*) (Shigeyoshi et al., 1997; Tei et al., 1997; Gekakis et al., 1998; Takumi et al., 1998; Kume et al., 1999; Shearman et al., 2000; Albrecht et al., 2001). PERs and CRYs accumulate in the cytoplasm throughout the day, but into the night, PER and CRY form heterodimers which translocate back into the nucleus and repress their own transcription *via* interaction with the BMAL1/CLOCK and/or BMAL1/NPAS2 complex. This process completes the TTFL and requires ∼24 h to complete a full cycle; review in (Partch et al., 2014). Notably, the degradation of PER and CRY proteins is regulated by casein kinase 1δ (CK1δ) and CK1ϵ, which ultimately determines the period length of the circadian clock, or the time it takes to complete a cycle (Etchegaray et al., 2009; Lee et al., 2011).

In addition to the primary feedback loop, several additional ancillary regulatory loops contribute to the circadian clockwork (Cox and Takahashi, 2019). Indeed, CLOCK/NPAS2:BMAL1 complexes also drive transcription of reverse-ErbA alpha and beta (REV-ERBα and REV-ERBβ; encoded by *Nr1d1* and *Nr1d2*, respectively), which compete with retinoic acid-related orphan receptors (RORα, RORβ and RORγ; encoded by *Nr1f1*, *Nr1f2* and *Nr1f3*, respectively) for binding at retinoic acid-related orphan receptor response elements (RRE) within the promoter regions of DNA (Preitner et al., 2002; Sato et al., 2004; Akashi and Takumi, 2005). By binding to RREs, RORs activate transcription while REV-ERBs inhibit transcription of BMAL1. REV-ERBs and RORs have also been shown to regulate CRY1 and NPAS2 in a similar fashion (Preitner et al., 2002; Crumbley et al., 2010). Notably, additional feedback loops have been described, including the D site of albumin promoter (albumin D-box) binding protein (DBP) feedback loop (Lopez-Molina et al., 1997; Ripperger et al., 2000; Yamaguchi et al., 2000), the basic helix-loop-helix protein E 40 (BHLHE40) and BHLHE41 loops (also known as DEC1 and DEC2) (Honma et al., 2002; Kawamoto et al., 2004; Nakashima et al., 2008), and the computationally highlighted repressor of the network oscillator (CHRONO) feedback loops (Anafi et al., 2014; Goriki et al., 2014; Ono et al., 2021). Altogether, these ancillary TTFLs allow for redundancy to reinforce the molecular clock’s rhythmicity and protect its function. Mutation or loss of most clock proteins can be compensated for to minimize interruption to rhythmicity. NPAS2, for example, can completely compensate for the loss of CLOCK function (Reick et al., 2001; Bertolucci et al., 2008; Landgraf et al., 2016b) as evidenced by the fact that double knockout of both *Clock* and *Npas2* is needed to completely abolish activity rhythms (DeBruyne et al., 2007). However, the only clock protein that *cannot* be compensated for is BMAL1 (McDearmon et al., 2006). Notably, beyond just regulating molecular clock function, the circadian molecular clock has far-reaching effects across the organism’s genome. Indeed, transcriptome-wide sequencing studies demonstrate that ∼40% of the rodent genome exhibits circadian oscillations, and 80% of genes are rhythmic in primates (Zhang et al., 2014; Mure et al., 2018). These genome-wide rhythms and subsequent protein rhythms are mediated through a host of transcriptional, post-transcriptional, translational, and post-translational mechanisms that in turn temporally organize nearly all aspects of cellular function, physiology, and ultimately behavior.

Taken together, the SCN has traditionally been considered the central pacemaker transducing environmental information (namely light) to generate and synchronize circadian rhythms from the system level down to the cellular circadian molecular clock level. However, other brain regions have recently been discovered that produce sustainable, entrainable 24-h rhythms in core clock expression, electrophysiological activity, and/or overall function, reviewed in (Dibner et al., 2010; Honma, 2018; Begemann et al., 2020). These potential extra-SCN oscillators point to a multi-oscillatory system in which other tissues, central or peripheral, have inherent circadian oscillatory properties that temporally organize physiology/function but are synchronized by the SCN to maintain overall circadian organization of the organism and its behaviors. While the SCN is important for entraining rhythms, it is thought that localized extra-SCN oscillators may play a more integral role in regulating specific behaviors across time of day. In this review, we will present evidence that suggests mesolimbic reward structures, specifically the ventral tegmental area (VTA) and nucleus accumbens (NAc), serve as extra-SCN circadian oscillators with a functional circadian clock and that this timekeeping system ultimately drives known rhythms in reward processing and motivated behaviors. Finally, we will discuss the functional implications of both the VTA and the NAc having circadian molecular clock function as it relates to drug abuse and the development of substance use disorders (SUDs).

## The Mesolimbic Reward Pathway and Substances of Abuse

Accumulating evidence from both clinical and preclinical studies points to the mesolimbic pathway as being the primary neural structures mediating reward and reward-related behaviors (Wise, 2008; Koob and Volkow, 2010; Baik, 2013; Volkow and Morales, 2015). Notably, there are two primary pathways that connect the midbrain to the striatum: the *nigrostriatal pathway*, consisting of dopaminergic projections from the substantia nigra (SN) to the dorsal striatum (DS), and the *mesolimbic pathway*, consisting of dopaminergic projections from the VTA to the NAc in the ventral striatum. While there is some evidence to suggest the nigrostriatal pathway may play some role in mediating reward (Wise, 2009), the mesolimbic pathway has historically been considered the central reward pathway of the brain. Briefly put, when dopaminergic neurons in the VTA are activated, dopamine is released from the VTA axon terminals into NAc synapses. The VTA-mediated activation of dopamine receptor-expressing neurons in the NAc is thought to underly the assigning of motivational/hedonic salience to a stimulus (e.g., natural rewards like food and socialization, or even non-natural rewards like drugs of abuse). This activation, in turn, reinforces rewarding behavior and ultimately drives goal-directed behaviors towards rewarding stimuli. (Schultz, 2002; Volkow and Morales, 2015; Morales and Margolis, 2017).

The VTA is a heterogeneous region containing dopaminergic, GABAergic, glutamatergic, and other neuronal subtypes. Though heterogeneous, dopaminergic neurons are the primary neuronal subtype, accounting for 60%–65% of all VTA neurons in rodent models (Swanson, 1982; Nair-Roberts et al., 2008). Historically, these dopamine neurons have primarily been classified by their expression of tyrosine hydroxylase (TH) (Grace and Onn, 1989; Nair-Roberts et al., 2008), a rate-limiting enzyme in the biosynthesis pathway for dopamine, as well as by their electrophysiological properties (Ungless and Grace, 2012). For the synthesis of dopamine, TH converts L-tyrosine into L-DOPA, which is then converted into dopamine by aromatic L-amino acid decarboxylase. In addition to expressing TH, dopaminergic neurons in the VTA generally also express dopamine transporter (DAT) and vesicular monoamine transporter 2 (VMAT2), which work to reuptake dopamine after synaptic release and package dopamine back into synaptic vesicles, respectively. However, it is important to note rodent studies have shown there are subpopulations of TH-expressing dopamine neurons that co-release glutamate (Stuber et al., 2010; Hnasko et al., 2012; Mingote et al., 2017) or even minimally co-express DAT and VMAT2 (Lammel et al., 2008; Stamatakis et al., 2013)—further underscoring the cellular heterogeneity of the VTA even within the defined neuronal subtype populations. However, one generally common feature among dopamine neurons in the VTA is their distinct electrophysiological firing patterns; dopamine neurons either fire in a stable tonic pattern of 1–8 Hz frequency or in a more transient high-frequency phasic firing pattern of >15 Hz frequency. This phasic high-frequency firing pattern is thought to result in the fastest and largest dopamine release into the NAc necessary to drive reward, while the tonic firing pattern is associated with less dopamine release and reward attenuation—both mediated by specific dopamine receptors. (Grace and Onn, 1989; Dreyer et al., 2010; Ungless and Grace, 2012; Paladini and Roeper, 2014).

Unlike the VTA, the NAc almost entirely consists of GABAergic medium spiny neurons (MSNs)—with rodent studies suggesting GABAergic MSNs account for roughly 95% of neurons in the NAc (Surmeier et al., 2007; Heiman et al., 2008; Gangarossa et al., 2013). Within this population, the MSNs can be divided into two primary functional subtypes based on their relative expression of either D_1_ (*Drd1*) or D_2_ (*Drd2*) G-protein coupled dopamine receptors. Though there are actually five specific dopamine receptor subtypes (D_1_–D_5_), each of the receptors are generally classified as being either D_1_-like receptors (D_1_ and D_5_) or D_2_-like receptors (D_2_–D_4_), based on whether they stimulate or inhibit secondary messenger cyclic AMP (cAMP), respectively (Missale et al., 1998; Surmeier et al., 2007; Bhatia et al., 2022). In addition to differences in synaptic plasticity, intrinsic excitability, and signaling cascades (Lu et al., 1998; Surmeier et al., 2007; Baik, 2013), the most notable difference between D_1_-receptor containing versus D_2_-receptor containing MSNs is their involvement in projections back to the VTA. In addition to receiving dopamine input from the VTA, the NAc sends reciprocal projections back to the VTA through both a direct *striatonigral* pathway (projecting to the VTA and substantia nigra in the ventral mesencephalon) and an indirect *striatopallidal* pathway (projecting to the ventral mesencephalon by way of the globus pallidus), with both having downstream effects on thalamus-mediated motivated motor control (Macpherson et al., 2014). Notably, the direct pathway is thought to primarily consist of D_1_-receptor containing MSNs and is reward-promoting, while the indirect pathway is thought to primarily consist of D_2_-receptor containing MSNs and aversion-promoting (Hikida et al., 2010, 2013; Lobo and Nestler, 2011; Kravitz et al., 2012). However, it is important to note recent evidence suggests this classification may be oversimplifying and ignoring nuances of the system (Kupchik et al., 2015), which should be taken into consideration. Finally, the NAc can also be subdivided anatomically into the lateral core and medial shell, with the core primarily mediating goal-directed behavior and learning and the shell primarily mediating processing/assigning of hedonic value and salience (Meredith et al., 2008; West et al., 2018). Differences between the shell and core arise through differences in integrating not only VTA dopamine input, but also differential glutamatergic input from the prefrontal cortex, hippocampus, thalamus, amygdala, and other regions (Scofield et al., 2016). Notably, the NAc is also is enriched with astrocytes, or astroglia, that serve an integral role in regulating these glutamatergic synapses, among many other functions (Scofield and Kalivas, 2014). Though the NAc integrates information from all these cortical and limbic structures to mediate motivation and goal-directed behaviors, the VTA-NAc projection is most integral to the processing of reward, hedonic value, and incentive salience.

The “Dopamine Hypothesis of Addiction” posits that substances of abuse, much like natural rewards, act directly on this reward system to increase VTA-mediated dopamine release into the NAc to both promote reward and drive subsequent reward-seeking behaviors (Spanagel and Weiss, 1999; Diana, 2011). Both clinical and preclinical studies have shown that nearly all substances of abuse produce an increase of dopamine and/or dopamine receptor binding in the NAc, including cocaine, amphetamine, opioids, alcohol, marijuana, and nicotine (Willuhn et al., 2010; Wise and Robble, 2020). In particular, it’s thought that drugs of abuse significantly increase phasic dopaminergic firing in the VTA that results in fast, large, and sustained supraphysiological releases of dopamine into the NAc shell that both activate reward-promoting D_1_ receptor containing MSNs of the direct pathway while inhibiting reward-attenuating D_2_ receptor containing MSNs of the indirect pathway (Di Chiara, 2002; Volkow et al., 2008; Owesson-White et al., 2009; Volkow and Morales, 2015). In addition to increasing dopamine levels, repeated drug exposure has also been shown to downregulate D_2_ receptor expression and/or binding (Volkow et al., 2001; Nader et al., 2006; Volkow et al., 2009; Trifilieff and Martinez, 2014), as well as induce long term neuroplasticity changes that enhance sensitivity of the NAc to substances of abuse and drive motivation/reward-seeking behaviors (Kalivas and O’Brien, 2008; Grueter et al., 2012; Volkow and Morales, 2015). Though significant advances have been made in understanding the transition from drug reward to compulsive drug seeking and SUDs (Volkow and Morales, 2015; Koob and Volkow, 2016; Poulton and Hester, 2020), the specific mechanisms that drive this transition are still unknown. Of particular interest for this review, disruption to circadian rhythms and/or circadian regulation of the VTA and NAc are mechanisms warranting further investigation (Logan et al., 2014, 2018; Becker-Krail and McClung, 2016; Webb, 2017; Tamura et al., 2021). More specifically, accumulating evidence suggests the VTA and NAc are not only hubs for reward regulation, but also serve as extra-SCN circadian oscillators.

## The Ventral Tegmental Area as a Circadian Oscillator

Through preclinical research, we have long appreciated that reward processing is intertwined with the circadian system (Parekh and McClung, 2015; DePoy et al., 2017). For example, food is a naturally potent reward across species, and its intake is highly governed by the circadian system such that food intake aligns with organisms’ active phase (Volkow et al., 2011; Challet, 2019; Mendoza, 2019; Pickel and Sung, 2020). This is also true for drugs of abuse; decades of preclinical work has revealed diurnal variations in drug reward sensitivity, conditioned place preference, locomotor sensitization, and operant self-administration, with behavior primarily peaking during the animal’s active phase (Webb et al., 2009; Webb et al., 2015). This is partly attributed to the observation that both natural rewards, such as food, and especially drugs of abuse, act as strong zeitgebers that entrain the circadian system and subsequent motivated behaviors (Gillman et al., 2019). Alternatively, accumulating evidence suggests both the VTA and NAc act as circadian oscillators themselves that regulate reward and motivation across time of day.

While it was long thought that VTA dopamine neurons do not exhibit circadian variation in activity, several studies in the past 2 decades suggest the contrary. Through electrophysiological recordings in anesthetized rats, VTA neurons exhibited a circadian rhythm in their spontaneous activity, with activity being greatest during the animal’s active phase or dark phase (Luo et al., 2008; Luo and Aston-Jones, 2009). Expression of cFos, a marker for neuronal activation, is also significantly higher in VTA TH+ and TH− neurons in the animal’s active phase or dark phase (Baltazar et al., 2013). In another study in anesthetized rats, VTA neurons exhibited intra-diurnal 12-h rhythmic patterns of firing across both the light and dark phases, as well as total active dopamine neurons and the pharmacological response of D_2_ receptors both higher in the dark phase (Domínguez-López et al., 2014). Finally, *in vivo* multi-unit activity recordings in mice have also revealed VTA activity to exhibit a strong circadian rhythm, with VTA neuronal activity significantly higher during the animal’s active phase (dark phase) (Fifel et al., 2018). Interestingly, however, the substantia nigra, a neighboring dopamine-rich midbrain region, did not exhibit this circadian variation in multi-unit activity (Fifel et al., 2018). Ultimately, the rhythmic activity of the VTA can be attributed to both local circadian molecular clock function and entrainment by indirect SCN innervation.

Several studies report diurnal variation of core circadian clock gene and protein expression in the VTA (Baird et al., 2013; Chung et al., 2014; Wang et al., 2019). This is further supported by *ex vivo* bioluminescent recordings of PERIOD2::LUCIFERASE rhythms in the VTA of PER2:LUC reporter mice (Landgraf et al., 2014; Logan et al., 2015; Landgraf et al., 2016a), a knock-in mouse line expressing the firefly *luciferase* gene fused to the *Period2* gene that allows for real-time visual monitoring of self-sustained circadian oscillations (Yoo et al., 2004)). Most notably, a recent study utilized whole-genome microarray hybridization analysis to investigate transcriptome-wide rhythms in mouse VTA samples collected across six times of day (ZT0, 4, 8, 12, 16, and 20) (Koch et al., 2020). Strikingly, the VTA transcriptome exhibited robust rhythms in gene expression; roughly 10% of the 2,643 transcripts investigated had significant rhythms with peaks in expression primarily clustering at ZT3 (inactive/light phase) and even more so at ZT16 (active/dark phase) (Koch et al., 2020). In particular, several studies have shown VTA dopamine-related genes diurnally vary in expression and function, and/or maybe directly regulated by the circadian molecular clock, including TH, DAT, and the dopamine degradation enzyme monoamine oxidase A (MAOA) (Figure 1B) (McClung et al., 2005; Hampp et al., 2008; Chung et al., 2014; Ferris et al., 2014; Logan et al., 2019; Alonso et al., 2021). Together, these findings point to a functional circadian clock system within the VTA that organizes its function on a 24-h timescale. While this local molecular clock works to temporally coordinate VTA gene expression and subsequent cellular physiology, these rhythms may be entrained by rewarding stimuli (e.g., natural rewards and drugs of abuse) and/or through indirect innervation by the SCN.

**FIGURE 2:**
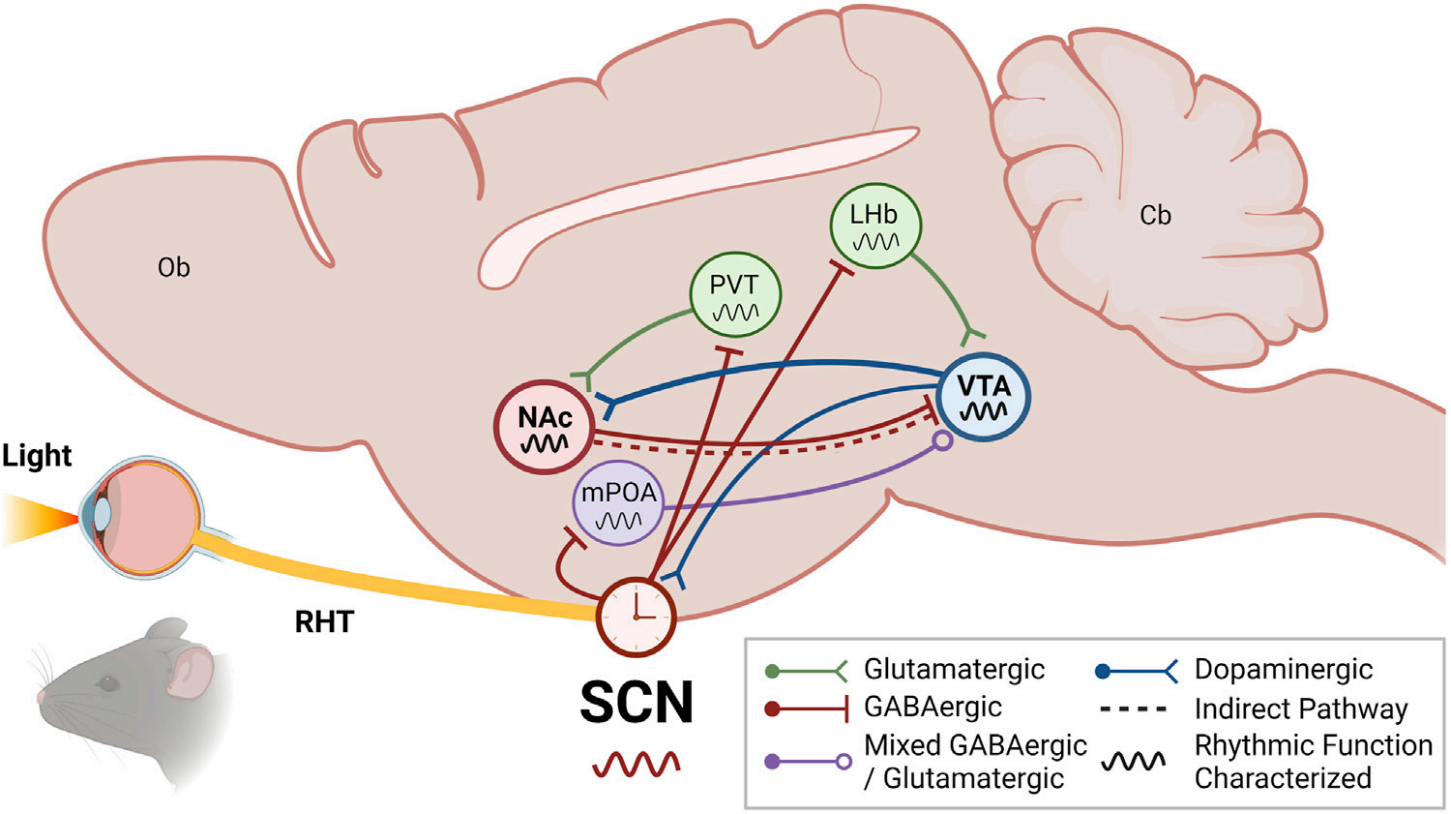
The VTA and NAc are integrated into the circadian system through indirect SCN input. The suprachiasmatic nucleus (SCN) of the anterior hypothalamus receives photic information from intrinsically photosensitive retinal ganglion cells (ipRGCs) in the retina *via* the retinohypothalamic tract (RHT). While the SCN generates rhythms through its highly coupled network of neuronal and glial oscillators, the SCN rhythms are entrained to environmental light/dark cycles by retinal photic information. The SCN synchronizes extra-SCN oscillators throughout the organism, both centrally and peripherally, *via* autonomic, metabolic, and hormonal signals. These extra-SCN oscillators are thought to afford local temporal control of complex physiology and behaviors. In particular, the ventral tegmental area (VTA) and nucleus accumbens (NAc) of the mesolimbic pathway may receive and integrate circadian information through indirect connections with the SCN. In addition to reciprocally communicating with the NAc through direct and indirect projections to mediate reward, the VTA may receive indirect SCN circadian input *via* the lateral habenula (LHb) and/or the medial preoptic area (mPOA)—both of which are direct outputs of the SCN and also receive photic information from ipRGC innervation. Notably, the VTA has also been shown to directly innervate the SCN and regulate photoentrainment. The NAc may receive indirect SCN circadian input *via* the paraventricular nucleus of the thalamus (PVT), one of the densest extrahypothalamic outputs of the SCN, or *via* the VTA. Schematic illustrates a sagittal section of a rodent brain. Undefined: olfactory bulb (Ob); cerebellum (Cb); See key for more details. Figure created with BioRender.com.

To date, no studies have identified a direct projection from the SCN to the VTA. However, some studies have reported the VTA can receive circadian information from the SCN *indirectly* by way of the medial preoptic area (mPOA) of the hypothalamus and the lateral habenula (LHb) (Figure 2). It is well established that the SCN sends direct projections to the mPOA and the LHb (Van Drunen and Eckel-Mahan, 2021). The POA is well established as a region regulating sleep/wake and other circadian rhythms through its innervation from both the SCN and ipRGCs directly (Rothhaas and Chung, 2021; Tsuneoka and Funato, 2021; Zhang et al., 2021). One of the first studies to show the SCN indirectly projects to the VTA, Luo and Aston-Jones utilized retrograde tracer transsynaptic pseudorabies virus injected into the VTA of rats and found labeling in the SCN that was indicative of an indirect afferent which was significantly abolished with mPOA lesion (Luo and Aston-Jones, 2009). This study confirmed the functional implications of this innervation through electrophysiological recordings showing VTA neuronal activity exhibits diurnal variation in impulse firing as a result of significantly higher rates during the active phase (Luo et al., 2008; Luo and Aston-Jones, 2009). Alternatively, this circadian timekeeping in the VTA may also be mediated through the LHb. Extensive work has shown the LHb exhibits robust, sustainable rhythms that are entrained by both innervation from the SCN and even direct photic input from the ipRGCs themselves (Zhang et al., 2009; LeGates et al., 2014; Sakhi et al., 2014; Baño-Otálora and Piggins, 2017; Mendoza, 2017). Moreover, the LHb is well-established to regulate reward and motivated behaviors through glutamatergic innervation of both dopaminergic and GABAergic VTA neurons, which can bidirectionally modulate VTA function depending on which neurons are being activated (Omelchenko et al., 2009; Quina et al., 2015; Baker et al., 2016; Wallace et al., 2020). Taken together, the LHb may serve as a key mediator between the SCN and the VTA, working to impart circadian timing and driving the known rhythms in VTA clock and reward function (Salaberry and Mendoza, 2015; Mendoza, 2017). However, future studies testing this idea are still needed.

Finally, in addition to the VTA’s known rhythmicity and indirect innervation from the SCN, a recent study in mice has uncovered a potential role for the VTA to reciprocally regulate the SCN and thus overall circadian rhythms. Though it has long been known that the D_1_ receptor is expressed in the SCN of both mice and non-human primates (Weaver et al., 1992; Bender et al., 1997; Rivkees and Lachowicz, 1997), the functional role of this expression has remained largely unknown. The importance of D_1_ receptor expression in the SCN was demonstrated using the *Drd1* knockout mouse model coupled with SCN site-specific manipulations and measuring photic entrainment (Grippo et al., 2017). Strikingly, *Drd1* global knockout mice exhibited a significantly slower rate of photoentrainment of behavioral rhythms that could be ameliorated through selective re-expression of *Drd1* specifically in the SCN. Most notably, using retrograde fluorescent bead tracing, a direct projection from the VTA to the SCN was identified for the first time, and when activated using excitatory Gq-coupled Designer Receptors Exclusively Activated by Designer Drugs (DREADDs), VTA-SCN projection neurons significantly accelerate circadian photoentrainment of behavioral rhythms in mice (Grippo et al., 2017). This relationship is even supported from earlier findings in which knock-down of the circadian gene *Clock* specifically in the VTA significantly reduced the circadian period and amplitude of mouse wheel-running rhythms (Mukherjee et al., 2010). In theory, this reciprocal communication between the VTA and SCN sets up a feedback mechanism by which reward processing and reward-related behavior is under circadian regulation, and conversely, rewarding stimuli (e.g., natural rewards or drugs of abuse) can directly affect circadian rhythms to temporally organize and drive reward-seeking behaviors. Although separate evidence points to this mechanism as being possible, a study tying together these observations has yet to be reported.

## The Nucleus Accumbens as a Circadian Oscillator

As mentioned, extensive preclinical work has illustrated the temporal organization of reward and reward-related behaviors with peak activity often aligning with individuals’ active phase (Logan et al., 2014; Parekh and McClung, 2015; Webb et al., 2015; Tamura et al., 2021). Alongside the role of the VTA in mediating this rhythmicity in reward, there is considerable evidence suggesting the NAc may also serve as a circadian oscillator that receives and integrates both sensory and circadian information to temporally regulate motivated behaviors. This is evidenced by the observation that the NAc exhibits diurnal variation in activity/function, robust rhythms in molecular clock function, and its integrated position within the circadian system.

Evidence from electrophysiological recordings suggest MSN activity in the NAc is under circadian regulation. Using *ex vivo* whole-cell patch-clamp recordings, Parekh et al. (2018) measured MSN excitatory synaptic transmission, synaptic strength, and intrinsic excitability in mice during both the light and dark phase. Interestingly, MSNs exhibit diurnal variability in glutamatergic synaptic transmission and intrinsic excitably, with peaks during the mouse’s active/dark phase (Parekh et al., 2018). In addition to NAc neuronal activity, nearly all aspects of dopamine handling and signaling have been shown to diurnally vary in the NAc presynaptically, postsynaptically, and extracellularly. Through studies in rodents, concentrations of dopamine and its metabolites have been shown to have robust diurnal rhythms in the NAc (Schade et al., 1995; Castañeda et al., 2004; Hampp et al., 2008; Ferris et al., 2014; Koch et al., 2020; Alonso et al., 2021). This rhythmicity in dopamine levels can be attributed to both the aforementioned rhythmic activity of the VTA, as well as diurnal variation in the proteins that regulate dopamine synthesis, degradation, and signaling. Work from the McClung lab and others have shown significant diurnal variation in the expression of all dopamine receptors in the NAc (e.g., D_1_, D_2_ and D_3_ receptors), as well as levels/activity of TH, DAT, and MAOA (Figure 1B) (Sleipness et al., 2007, 2008; Hampp et al., 2008; Chung et al., 2014; Ferris et al., 2014; Ozburn et al., 2015; Logan et al., 2019; Alonso et al., 2021). Moreover, at the gene level, transcription of *Th*, *Dat*, *Maoa*, and *Drd1,2,3* have actually shown to be directly regulated by the circadian molecular clock (e.g., either demonstrated through knockdown studies or through identifying E-boxes and/or RREs in their promoters) (Ueda et al., 2005; Hampp et al., 2008; Ikeda et al., 2013; Chung et al., 2014; Ozburn et al., 2015; Logan et al., 2019). This regulation by the circadian molecular clock is important given that the NAc has robust rhythms in molecular clock function.

Studies in both rodents and human post-mortem tissue have shown significant rhythmicity in NAc circadian molecular clock function. Using the PER2:LUC mouse model, sustained bioluminescent rhythms have been detected in the NAc *ex vivo* (Logan et al., 2015; Landgraf et al., 2016a; Porcu et al., 2020). This corroborates gene expression analyses in mice showing significant diurnal variation in molecular clock mRNA levels in the NAc (Falcon et al., 2013; Natsubori et al., 2014; Logan et al., 2015; Brami-Cherrier et al., 2020; Porcu et al., 2020; Becker-Krail et al., 2022b), as well as protein expression analyses in the striatum (Schnell et al., 2015). This is also seen in human post-mortem NAc tissue RNA-sequencing analyses where transcript levels are organized by time-of-death and tested for rhythmicity. In the NAc of healthy neurotypical control donors, robust rhythms in the canonical clock genes have been seen (e.g., *Bmal1*, *Npas2*, *Period*, *Cryptochrome*, and *Rev-erbα*), as well as pathway analyses revealing *Circadian Rhythm Signaling* as a pathway enriched among the top rhythmic genes (Li et al., 2013; Ketchesin et al., 2021; Xue et al., 2022). Finally, though these findings point to a functional molecular clock at the whole NAc level, functional investigation of these rhythms has largely been in MSNs or MSN subtypes. Remarkably, in a study published just this year, NAc astrocytes in particular were also found to have robust rhythms (Becker-Krail et al., 2022a). Using next-generation total RNA-sequencing in NAc astrocytes across time-of-day, NAc astrocytes were found to not only express robust rhythms in all the canonical clock genes, but also roughly 43% of the entire NAc astrocyte transcriptome exhibited a significant diurnal rhythm (Becker-Krail et al., 2022a). Relative to only 6% detected to be rhythmic across the whole NAc (all cells) (Brami-Cherrier et al., 2020), this newly characterized astrocyte-specific rhythmicity highlights the complex nature of circadian regulation in the NAc and underscores the need to investigate circadian regulation of reward regions in a cell-type-specific manner, including both neurons and glia. Altogether, these studies point to a self-staining, functional circadian clock system within the NAc that organizes its function across time of day. Whereas this local molecular clock works to temporally coordinate NAc gene expression and subsequent cellular physiology, much like the VTA, these rhythms may also be entrained by rewarding stimuli (e.g., natural rewards and drugs of abuse) and/or through indirect innervation by the SCN.

In common with the VTA, a direct projection from the SCN to the NAc remains unspecified. However, in addition to the direct input from the VTA that may impart rhythmicity, some studies have reported the NAc may receive circadian information from the SCN *indirectly* by way of the paraventricular nucleus of the thalamus (PVT) (Figure 2). The PVT is a highly rhythmic thalamic nucleus that reciprocally communicates with the SCN, receiving one of the densest extrahypothalamic direct outputs of the SCN while also sending glutamatergic projections back (Kolaj et al., 2012; Colavito et al., 2015). The PVT has been implicated in regulating mood, reward, stress, sleep/wake, and arousal, in part due to its interface with the SCN and a number of corticolimbic regions, including the PFC, amygdala, and the NAc (Moga et al., 1995; Li and Kirouac, 2008; Vertes and Hoover, 2008). Though only the amygdala has been confirmed to receive indirect SCN input *via* the PVT using tracing techniques (Peng and Bentivoglio, 2004), it is likely this also extends to the NAc given the NAc’s aforementioned rhythmicity in function and the known role of PVT-NAc projections in mediating reward (Kirouac, 2015; Barson et al., 2020; De Groote and de Kerchove d’Exaerde, 2021). Furthermore, PER expression is similar between the NAc and PVT in rodents, with higher expression in the active/dark phase, and in both regions is entertainable to food intake (Angeles-Castellanos et al., 2007; Feillet et al., 2008; Falcon et al., 2013). However, whether the PVT relays SCN photic information or rhythmicity to the NAc and whether it may be important for reward remains to be investigated.

Finally, in addition to the role of the circadian system in regulating NAc function, accumulating preclinical evidence suggests the NAc may in turn also regulate circadian rhythms, namely sleep/wake rhythms. Though it has long been known that many psychiatric and neurological disorders commonly share both disruptions in sleep and altered NAc function, including drug abuse and SUDs (Wulff et al., 2010; Russo and Nestler, 2013; Ahrens and Ahmed, 2020), we have only more recently begun to appreciate the role the NAc plays in regulating sleep/wake. Through electroencephalography (EEG) studies in rats, rats with generalized lesions to the NAc core or shell exhibited 26% and 17% increases in wakefulness, respectively, and reductions in non-rapid eye movement (NREM) sleep bout duration (Qiu et al., 2010, Qiu et al., 2012). Expanding on these findings, optogenetics or DREADDs were used to target a specific NAc MSN subpopulation and investigate its role in sleep (Oishi et al., 2017). Activation of NAc core adenosine A_2a_ receptor-expressing MSNs of the indirect pathway strongly promoted and increased slow-wave sleep (SWS), while inhibition of these neurons decreased SWS (Oishi et al., 2017; Valencia Garcia and Fort, 2018). Conversely, other studies have found optogenetic activation of VTA terminals in the NAc actually decreases NREM and REM in mice and can promote arousal (Eban-Rothschild et al., 2016). More specifically, using *in vivo* fiber photometry coupled with optogenetics or DREADDs, activation of D_1_ MSNs in the NAc rapidly induces a transition from NREM to wakefulness, while inactivation of these neurons suppresses arousal and increases nest-building (Luo et al., 2018). This differential regulation of sleep by the NAc is further supported by recent data showing activation/inactivation of D_1_ versus D_2_ MSNs can bidirectionally regulate sleep; i.e., *inactivation* of D_1_ MSNs or *activation* of D_2_ MSNs promotes sleep (D_2_ MSNs specifically promoting SWS), while *activation* of D_1_ MSNs promotes wake or arousal but targeting D_2_ MSNs has no effects on arousal (McCullough et al., 2021). However, beyond this role in differentially regulating sleep/wake rhythms, whether the NAc extends regulation to the broader circadian system is still unknown.

## Implications for Reward and Substance Abuse

During the past 2 decades, accumulating evidence points to an association between disruptions in circadian rhythms, drug abuse, and the development of SUDs. Nearly all substances of abuse have been shown to disrupt circadian rhythms (Bolelli et al., 1979; Vescovi et al., 1992; Danel et al., 2001). Those with SUDs also tend to have poor sleep parameters and exhibit significantly disrupted sleep/wake rhythms (Angarita et al., 2016; Koob and Colrain, 2020). Alternatively, individuals with disrupted circadian rhythms and/or poor sleep have an increased propensity to abuse substances and show altered reward-related brain function (Breslau et al., 1996; Brower et al., 2001; Johnson and Breslau, 2001; Hasler et al., 2012a, Hasler et al., 2012b; Hasler and Clark, 2013; Angarita et al., 2016; Dolsen and Harvey, 2017, Hasler et al., 2017; Logan et al., 2018; Goodhines et al., 2019). Together, these observations suggest a bidirectional relationship between circadian rhythm disruption and substance abuse, setting up the potential for a feed-forward, self-perpetuating cycle that could both establish and reinforce a SUD (Tamura et al., 2021). At the heart of this relationship, extensive preclinical evidence suggests proper circadian molecular clock function in both the VTA and NAc is integral in the regulation of reward and reward-related behavior.

In the VTA, genetic knockout and/or functional mutation studies have illuminated the functional significance of VTA circadian molecular clock function for reward-regulation and overall VTA function. One of the most studied models illustrating this, *ClockΔ19* mice carry a functional mutation in the transactivation domain of *Clock,* which disrupts its core clock binding functions (King et al., 1997). Interestingly, in addition to altered circadian function, *ClockΔ19* mice exhibit a hyper-hedonic behavioral phenotype; e.g., increased locomotor response to novelty, increased exploratory drive, increased cocaine preference, cocaine self-administration, and cocaine reward sensitivity, increased sucrose preference, and increased propensity for ethanol consumption (McClung et al., 2005; Roybal et al., 2007; Ozburn et al., 2012; Ozburn et al., 2013). Importantly, many aspects of the *ClockΔ19* phenotype can be recapitulated through a site-specific knockdown of *Clock* in the VTA (Mukherjee et al., 2010; Ozburn et al., 2013) and aspects of the reward-related phenotype are attributed to CLOCK regulating VTA dopamine neuron firing, ion channel expression, and importantly, diurnal rhythms in T*h* expression (Mukherjee et al., 2010; Logan et al., 2019). In fact, diurnal rhythms of *Th* expression are directly regulated by both CLOCK and REV-ERBα, whereby both circadian proteins normally act to repress transcription of *Th* in a phase-dependent manner through competitive binding at E-box and RREs in the *Th* promoter, respectively; however, knockdown or loss of either *Clock* or *Rev-erbα* in the VTA drives increased expression of *Th*, leading to disrupted diurnal rhythms in the VTA, higher NAc concentration of dopamine and metabolites, and increased reward-related behaviors (Chung et al., 2014; Logan et al., 2019). Notably, other clock proteins have been shown to regulate circadian expression and function of MAOA in the VTA (e.g., BMAL1, NPAS2 and PER2), and in *Per2* mutant mice, MAOA rhythms are blunted in the VTA leading to an accumulation of dopamine in the NAc and altered mood-related behaviors (Hampp et al., 2008). Together, these clock-disruption-mediated changes to VTA function and downstream behavior are particularly alarming given that substances of abuse have been shown to not only alter molecular clock rhythms specifically in the VTA (Li et al., 2009), but also entrain overall circadian rhythms (Kosobud et al., 2007; Honma and Honma, 2009; Glass et al., 2012; Prosser et al., 2014; Stowie et al., 2015; Gillman et al., 2019), which could feed into the VTA and/or partly be mediated by the VTA.

Relative to the VTA, additional evidence exists to suggest disruptions in NAc circadian molecular clock function drives aberrant reward-processing, motivation, and mood. Of particular interest for NAc-mediated reward regulation, NPAS2 is a circadian molecular clock protein that is highly expressed in the forebrain and enriched particularly in the D_1_ MSNs of the NAc (Zhou et al., 1997; Garcia et al., 2000; Reick et al., 2001; Ozburn et al., 2015), unlike the ubiquitous expression of its functional homolog CLOCK. Notably, while CLOCK seems to play a role in regulating reward through its expression in the VTA, NPAS2 has been shown to regulate reward through its expression in the NAc. The use of *Npas2* mutant mice or knockdown of *Npas2* in the NAc decreases cocaine conditioned place preference through its expression in D_1_ MSNs (Ozburn et al., 2015; Parekh et al., 2019; Becker-Krail et al., 2022b); *Npas2* mutant mice actually show significant increases in locomotor response to novelty, exploratory drive, cocaine self-administration and self-administration motivation (Ozburn et al., 2017; DePoy et al., 2020). In addition to its enriched expression in the D_1_ MSNs of the NAc, this disrupted reward regulation can also be attributed to NPAS2’s transcriptional regulation of reward-related transcripts and downstream regulation of excitatory synaptic transmission and plasticity (Parekh et al., 2019; Becker-Krail et al., 2022b). In addition to NPAS2, loss of function or decreased expression of other molecular clock proteins in the NAc (e.g., CLOCK, PER, CRY, and REV-ERBα) have also been shown to directly alter a whole range of behaviors, including reward, anxiety, cognitive function, stress-susceptibility, mood and depressive-like behaviors (De Bundel et al., 2013; Spencer et al., 2013; Landgraf et al., 2016a; Parekh et al., 2018; Zhao and Gammie, 2018; Porcu et al., 2020). Most notably, circadian molecular clock function specifically in NAc astrocytes is also critical for NAc function and reward regulation. In addition to NAc astrocytes exhibiting robust transcriptome-wide rhythms, mice with a loss of NAc astrocyte molecular clock function exhibit disrupted diurnal variation in reward behavior driven by increased light-phase locomotor response to novelty, exploratory drive, and food self-administration and motivation (Becker-Krail et al., 2022a). Alongside these behavioral effects, loss of NAc astrocyte molecular clock function also disrupts metabolic homeostasis in the NAc and alter glutamatergic synaptic transmission onto neighboring MSNs (Becker-Krail et al., 2022a). In addition to regulating reward-related behavior, altering but not disrupting NAc glial molecular clock function (i.e., *Per2* deletion) is sufficient to alter mood-related behavior through reducing behavioral despair (Martini et al., 2021). Finally, although preclinical findings suggest disrupted NAc molecular rhythms are associated with altered reward and drug abuse, whether molecular rhythms are disrupted in brains of people with SUD is largely understudied. In a preliminary report using human post-mortem NAc tissue from healthy donors and individuals previously diagnosed with opioid use disorder, total RNA sequencing analysis of the transcriptome was organized by time-of-death and uncovered robust molecular rhythms in the NAc that were significantly altered in those with opioid use disorder (Xue et al., 2022). Though it is unclear what functional role these rhythm alterations contributed to the development of the donor’s opioid use disorder, this is some of the first evidence in humans to suggest altered NAc molecular rhythms are associated with SUDs.

Interestingly, in addition to altering rhythmic function of the VTA and NAc, some evidence suggests substances of abuse may even generate circadian rhythms independent of the SCN. In rodents, exposure to methamphetamine, a widely-abused illicit psychostimulant, has been shown to induce robust circadian rhythms in locomotor activity (Honma et al., 1987). In this original study, SCN-lesioned rats were given methamphetamine in their drinking water across a range of concentrations and their locomotor activity was monitored. Strikingly, exposure to methamphetamine manifested a robust locomotor activity rhythm that was independent of the SCN central pacemaker (Honma et al., 1987). This methamphetamine-sensitive circadian oscillator (MASCO) has not only been shown to be independent of the SCN (Honma et al., 1987; Tataroglu et al., 2006; Honma and Honma, 2009), but may also be independent of the canonical circadian molecular clock mechanism, in that multiple clock gene knock-out mice still show methamphetamine induced rhythms in locomotor activity (Mohawk et al., 2009). Though potentially not mediated by the molecular clock, clock gene rhythms in extra-SCN regions *do* appear to correlate with methamphetamine induced behavioral rhythms and are desynchronized from SCN driven rhythms (Masubuchi et al., 2000; Natsubori et al., 2013) While the exact mechanisms are still unknown, this MASCO may be a derivative of dopaminergic ultradian oscillator (DUO) rhythms, whereby ultradian rhythms in locomotor activity (∼4 h) undergo period-lengthening to ∼24 h *via* a DAT and/or dopamine-mediated mechanism (Blum et al., 2014). Notably, the exact origin of this extra-SCN MASCO still remains to be determined. Given that methamphetamine primarily acts on dopamine neurons in the VTA to drive increased dopamine release and reduced uptake in the NAc, it is likely the mesolimbic system is involved in the MASCO. However, future studies should integrate the more recent circadian work in the VTA and NAc to specifically test a potential role for the mesolimbic system in MASCO manifested rhythms.

Taken together, the above studies not only highlight the presence of a circadian time-keeping system in the VTA and NAc, but also underscore the functional importance of an intact circadian molecular clock for the regulation of reward-processing and reward-related behaviors. Furthermore, these studies point to a potential mechanism by which circadian molecular clock disruption may drive drug abuse and eventual SUD development. However, given that most of the preclinical literature utilized molecular clock genetic mouse models and/or viral mediated-knock down of circadian molecular clock proteins to investigate circadian rhythm disruption, more translational models are needed in the study of circadian rhythms and reward-regulation. For example, exposure to light at night (LAN) has become increasingly pervasive with the advent of modern lighting, smart devices, and night shiftwork. Notably, in mice, LAN not only disrupts circadian rhythms, but also alters metabolism, immune function, stress, anxiety-like behavior, and mood-related behaviors (Fonken et al., 2010; Bedrosian et al., 2013; Fonken et al., 2013; Fonken and Nelson, 2014; Russart and Nelson, 2018; Walker et al., 2020a, Walker et al., 2020b; Bumgarner and Nelson, 2021, Walker et al., 2021). However, more translational models of circadian rhythm disruption such as LAN, or even social jet lag and shift work (Wittmann et al., 2006; Barclay et al., 2012; Opperhuizen et al., 2015; Haraguchi et al., 2021; Oneda et al., 2022), have been heavily underutilized in the study of circadian rhythm disruption and drug abuse, especially in the context of VTA and NAc circadian function. Future studies would benefit from utilizing these models to understand how unhealthy light practices (e.g., LAN, social jet lag, night shift work, etc.) not only affects circadian rhythms globally, but also how they specifically affect VTA and NAc function as they relate to drug abuse and vulnerability to develop SUDs.

## Conclusion

Though the SCN was considered to be the only central pacemaker or clock in the brains of mammals, emerging evidence suggests many extra-SCN circadian oscillators exist in the brain to drive rhythms in physiology and behavior (Begemann et al., 2020). Importantly, for a region to be considered an extra-SCN circadian oscillator, the region must 1) exhibit endogenous, self-sustaining, near 24 h rhythms that persist in the absence of environmental cycles and in isolation from all other tissues (e.g., *ex vivo* slice PER2:LUC rhythms), 2) be cable of being entrained (e.g., zeitgebers such as light, drugs of abuse, and other rewards), 3) be capable of communicating or transducing temporal information downstream, and 4) be temperature compensating or unaffected by temperature changes, though less relevant for homothermic animals like mammals with stable internal brain and body temperatures. (Guilding and Piggins, 2007). Following the identification of the retina as the first true extra-SCN oscillator (Tosini and Menaker, 1996), this opened the door to investigating oscillatory potential in regions across the entire brain (Begemann et al., 2020). In this review, we presented the most recent evidence in support of the VTA and NAc as extra-SCN circadian oscillators. In summary, the VTA and NAc both exhibit highly robust 24-h rhythms in function, electrophysiological activity, molecular clock function, and even transcriptome-wide rhythms. Notably, molecular clock function as measured by *ex vivo* PER2:LUC bioluminescent rhythms illustrates the VTA and NAc can sustain rhythms even in the absence of environmental cycles or input from other tissues, albeit for far fewer cycles than the SCN though. Moreover, rhythms of both the VTA and NAc are capable of being entrained, even by drugs of abuse or other rewarding stimuli. Finally, both the VTA and NAc have been shown to convey circadian information downstream and even directly affect circadian rhythms, with the VTA directly affecting photic entrainment and the NAc bidirectionally regulating sleep/wake. Not only do the VTA and NAc have intrinsic circadian oscillatory properties, but also extensive preclinical data suggests this circadian function is indeed integral to reward regulation and reward-related behaviors. Taken together, this review supports the VTA and NAc being classified as extra-SCN circadian oscillators, at least as semi-autonomous oscillators. However, further research is needed into characterizing the VTA and NAc as circadian oscillators in a cell-type-specific manner and through using cutting-edge genetic tracing tools. Additional studies should also further investigate 1) the relationship between the VTA, NAc and SCN as it relates to timekeeping, 2) where the VTA and NAc sit among the hierarchical multi-oscillatory network, and 3) how altogether these interactions facilitate reward and/or may go awry in SUDs. Such studies will provide invaluable information in the pursuit of novel therapeutic targets and the development of much needed new treatment options for SUDs.
